# Biallelic correction of sickle cell disease‐derived induced pluripotent stem cells (iPSCs) confirmed at the protein level through serum‐free iPS‐sac/erythroid differentiation

**DOI:** 10.1002/sctm.19-0216

**Published:** 2020-02-07

**Authors:** Juan J. Haro‐Mora, Naoya Uchida, Selami Demirci, Qi Wang, Jizhong Zou, John F. Tisdale

**Affiliations:** ^1^ Cellular and Molecular Therapeutics Branch National Heart Lung and Blood Institutes (NHLBI)/National Institute of Diabetes and Digestive and Kidney Diseases (NIDDK), National Institutes of Health (NIH) Bethesda Maryland; ^2^ iPS Cell Core Facility National Heart, Lung, and Blood Institute, National Institutes of Health Bethesda Maryland

**Keywords:** genome editing, hematopoiesis, hESCs, iPSCs, serum‐free, sickle cell disease

## Abstract

New technologies of induced pluripotent stem cells (iPSCs) and genome editing have emerged, allowing for the development of autologous transfusion therapies. We previously demonstrated definitive β‐globin production from human embryonic stem cell (hESC)‐derived erythroid cell generation via hemangioblast‐like ES‐sacs. In this study, we demonstrated normal β‐globin protein production from biallelic corrected sickle cell disease (SCD) iPSCs. We optimized our ES/iPS‐sac method for feeder cell‐free hESC maintenance followed by serum‐free ES‐sac generation, which is preferred for electroporation‐based genome editing. Surprisingly, the optimized protocol improved yields of ES‐sacs (25.9‐fold), hematopoietic‐like spherical cells (14.8‐fold), and erythroid cells (5.8‐fold), compared with our standard ES‐sac generation. We performed viral vector‐free gene correction in SCD iPSCs, resulting in one clone with monoallelic and one clone with biallelic correction, and using this serum‐free iPS‐sac culture, corrected iPSC‐generated erythroid cells with normal β‐globin, confirmed at DNA and protein levels. Our serum‐free ES/iPS‐sac protocol with gene correction will be useful to develop regenerative transfusion therapies for SCD.

## Graphical abstract

We have successfully corrected the sickle mutation in induced pluripotent stem cells (iPSCs) derived from a sickle cell disease patient using an improved serum‐free method for the generation of ES/iPS‐sacs, a hemangioblast‐like structure containing hematopoietic stem and progenitor cells that can be differentiated into erythroid cells containing mostly definitive globins, including γ‐ and β‐globins.

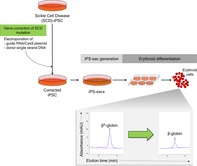

**Significance statement**

The sickle mutation in induced pluripotent stem cells (iPSCs) derived from a patient with sickle cell disease has successfully been corrected using an improved serum‐free method for the generation of ES/iPS‐sacs, a hemangioblast‐like structure containing hematopoietic stem and progenitor cells that can be differentiated into erythroid cells containing mostly definitive globins, including γ‐ and β‐globins.

## 1 INTRODUCTION

The development of human embryonic stem cells (hESCs) and induced pluripotent stem cells (iPSCs)^1^ has spurred rapid progress in the fields of stem cell biology and regenerative medicine. ES/iPSC technology is a powerful tool for disease modeling and drug discovery,^2^ improving speed, reliability, and specificity in the development of new therapies.^3^ ES/iPSCs could potentially serve as an alternative source of (a) red blood cells (RBCs) and platelets for transfusion, and (b) hematopoietic stem/progenitor cells (HSPCs) for transplantation. RBC transfusion from healthy donors is well established to treat acute and chronic anemia, but it carries the risks of immune‐based hemolysis, infectious disease transmission, and rarely graft‐vs‐host disease, in addition to issues concerning donor screening and blood collection.^4^ An in vitro culture system for RBC generation from CD34+ HSPCs and ES/iPSCs is being developed for an alternative RBC source.^5^, ^6^, ^7^, ^8^ Primary human CD34+ cells can be efficiently differentiated into erythroid cells in vitro,^5^ but CD34+ HSPCs must be collected prior to each differentiation culture since they lose self‐renewal abilities after differentiation. In contrast, ES/iPSCs can undergo large‐scale expansion followed by erythroid cell differentiation, making them a better candidate for an alternative RBC source.^6^, ^7^, ^8^ Autologous RBC transfusion could minimize the risk of alloimmunization by using erythroid cells differentiated from patient‐generated iPSCs that have been genetically modified. Therefore, transfusion of RBCs generated from autologous, gene‐corrected iPSCs would be an ideal therapeutic option for hereditary anemia, including sickle cell disease (SCD).

SCD is caused by a point mutation in the β‐globin gene (HBB:c.20A>T), which induces hemoglobin polymerization in hypoxic conditions, causing RBCs to become deformed and lead to symptoms such as hemolytic anemia, vaso‐occlusion, pain crisis, multiorgan damage, and early mortality.^9^ Since SCD is the most common single gene disorder, it is an attractive target for gene therapy approaches, such as gene correction. In the last decade, genome editing tools such as zinc finger nucleases (ZFNs) have been developed that allow for site‐specific DNA breakage.^10^ Correction of the SCD mutation has been reported in SCD iPSCs using ZFNs,^11^, ^12^ demonstrating normal β‐globin production at RNA levels when differentiated in vitro into erythroid cells^11^ and teratoma formation following mouse injection.^12^ The clustered regularly interspaced short palindromic repeats (CRISPR)/CRISPR associated protein 9 (Cas9) system was recently developed for site‐specific DNA cleavage, improving the efficiency, ease of design, and feasibility of genome editing.^13^, ^14^ In the CRISPR/Cas9 system, a guide RNA is utilized for the delivery of the Cas9 endonuclease to a specific DNA region, such as a disease‐related mutation site. Following delivery, the guide RNA and Cas9 protein complex cleave the DNA at the target site, leaving the opportunity for either non‐homologous end‐joining or homology directed repair to occur. Efficient, site‐specific DNA breakage at the mutated target site increases the frequency of homology directed repair when a normal donor DNA sequence is provided along with guide RNA and Cas9 protein, allowing for gene correction.

The combination of iPSC technology and CRISPR/Cas9‐based gene correction could be used as a possible regenerative medicine strategy for treating SCD. This approach has been recently described by using a guide RNA/Cas9 ribonucleoprotein (RNP) and an adeno‐associated virus type 6 (AAV6) donor vector demonstrating a biallelic correction of the SCD mutation with normal β‐globin production at the RNA expression level^15^; however, β‐globin protein production was not confirmed. The iPSC‐derived RBC generation is currently limited by insufficient expansion of erythroid cells per iPSC, primitive globin production (ζ‐ and ε‐globins), and reliance on xeno‐materials such as mouse feeder cells and fetal bovine serum (FBS). Takayama et al developed a 15‐day protocol using culture media containing FBS and vascular endothelial growth factor (VEGF) to produce functional megakaryocytes from hESCs through the formation of hESC‐derived‐sacs (ES‐sacs),^16^ which are hemangioblast‐like structures composed of hematopoietic‐like spherical cells, containing a CD34+CD45+ HSPC population, and endothelial‐like extracellular cells. We previously demonstrated that ES‐sac‐derived spherical cells can be differentiated into definitive erythroid cells that produce mainly γ‐globin and β‐globin (with low levels of ε‐globin) at both the RNA and protein levels.^8^, ^17^ The current understanding is that definitive RBCs (producing γ‐globin and β‐globin) are primarily generated by HSPCs, whereas primitive RBCs (producing ε‐globin) are derived from hemangioblast precursor cells in yolk sacs.^18^ We hypothesized that this ES‐sac‐based erythroid differentiation method would allow us to generate definitive erythroid cells from gene‐corrected SCD iPSCs. In addition, we sought to generate hESC‐derived definitive HSPCs/RBCs via ES‐sacs using serum‐free media, since it would reduce variability due to different FBS lots and would represent an important step toward xeno‐free clinical application.

Here, we optimized the ES‐sac generation protocol using feeder cell‐free hES/iPSC maintenance, a preferable method for electroporation‐based gene correction, followed by a serum‐free ES‐sac culture. The optimized protocol improved the yields of hematopoietic‐like spherical cells as well as β‐globin‐producing erythroid cells from hES/iPSCs. We demonstrated viral vector‐free biallelic β‐globin gene correction in SCD iPSCs followed by erythroid differentiation, which allowed for normal β‐globin production at the protein level.

## 2 MATERIALS AND METHODS

### 2.1 Cell culture conditions for cell lines, hESCs, and iPSCs

Human ESC line H1 (WiCell, Madison, Wisconsin) and an iPSC line (SCD‐iPS) previously established by our group from bone marrow stromal cells obtained from a SCD patient^8^ were maintained as undifferentiated cells on Corning Matrigel Growth Factor Reduced Basement Membrane Matrix (MT; Corning, New York) using mTeSR1 media (Stem Cell Technologies, Vancouver, British Columbia, Canada) in 37°C with 5% CO_2_. Culture media was changed daily, except for on weekends (Saturday and Sunday), and the cells underwent passage every 7 days using EDTA.^19^

A second group of H1 hESCs were maintained on irradiated primary Murine Embryonic Feeder cells (Thermo Fisher Scientific, Waltham, Massachusetts, http://www.thermofisher.com/) as described previously.^8^, ^17^ Media was changed daily, except on Sundays, and the cells underwent passage every 7 days using 1 mg/mL of collagenase IV (Thermo Fisher Scientific).

A mouse mesenchymal C3H10T1/2 cell line (American Type Culture Collection [ATCC], Manassas, Virginia, http://www.atcc.org/) was maintained in Basal Medium Eagle (BME, Life Technologies) supplemented with 10% FBS (Thermo Fisher Scientific) and 2 mM l‐glutamine (Thermo Fisher Scientific). The OP9 mouse bone marrow stromal cell line (ATCC) was maintained in Minimum Essential Medium alpha no nucleosides (MEM alpha, no nucleosides; Life technologies) supplemented with 20% FBS. Both C3H10T1/2 and OP9 cells were irradiated (50 Gy) before being used as feeder cells.

### 2.2 hES/iPSC‐derived erythroid cell differentiation through ES/iPS‐sacs

**Figure 1:**
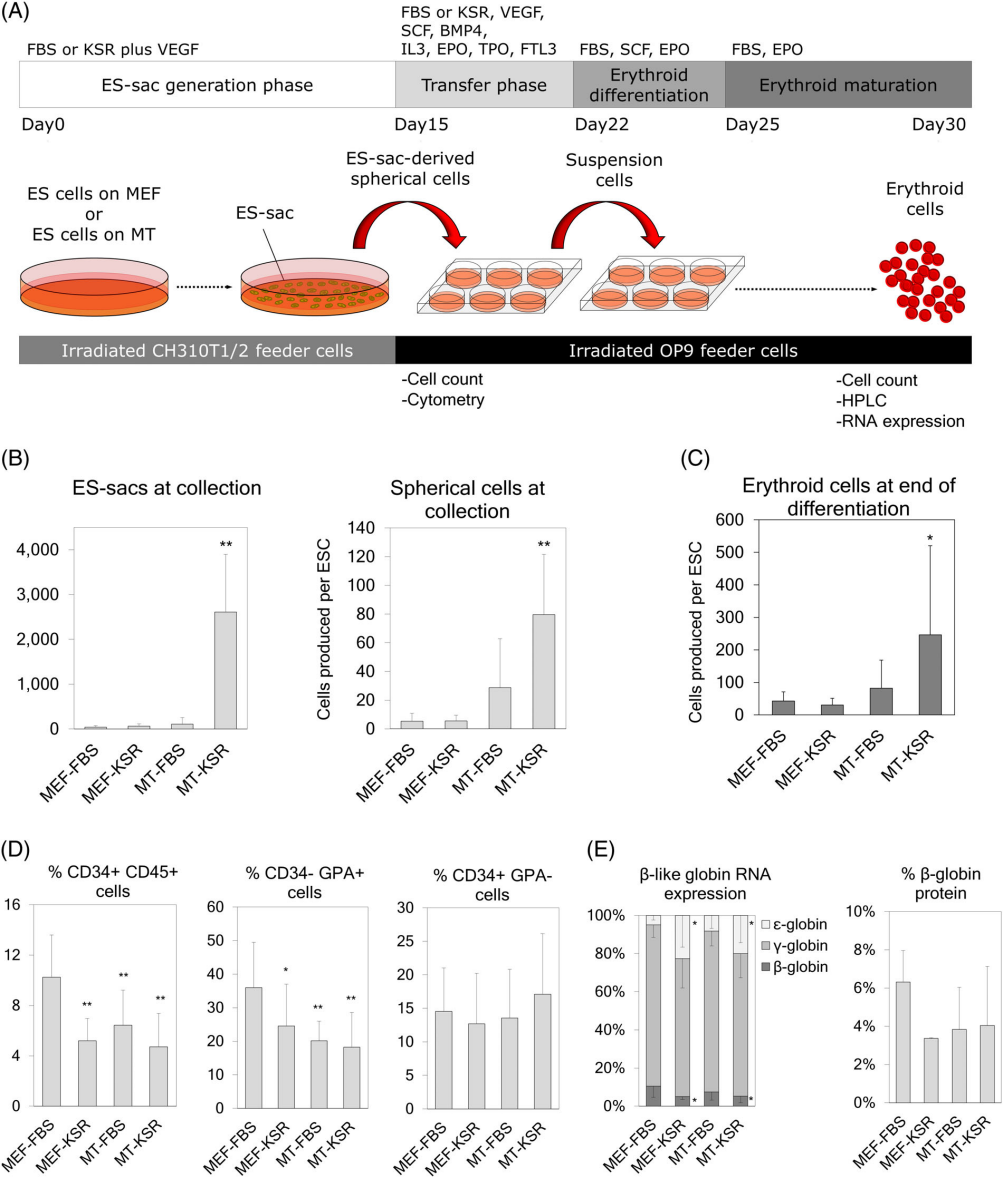
A, ES‐sac generation from feeder cell‐on or feeder cell‐free human embryonic stem cells (hESCs). ES‐sacs were generated with serum‐containing or serum‐free media, and hematopoietic‐like spherical cells were differentiated into erythroid cells. B, Left panel: Yield of ES‐sacs generated per culture dish at day 15 using four different conditions (MEF‐FBS, MEF‐KSR, MT‐FBS, and MT‐KSR). Right panel: Yield of ES‐sac‐derived spherical cells per hESC at day of collection (day 15). C, Yield of erythroid cells produced per hESC during erythroid differentiation. D, Percentages of cell surface markers in ES‐sac‐derived spherical cells. E, β‐like globin production at the protein level in ES‐sac‐derived erythroid cells, analyzed by RP‐HPLC. Data reported as mean ± SD. Statistical analysis was performed by Dunnett's test, compared with the MEF‐FBS group (*P < .05 and **P < .01). MEF‐FBS, n = 3; MEF‐KSR, n = 3; MT‐FBS, n = 11; MT‐KSR, n = 15 for all analysis except for RNA expression (Figure 1E left: n = 2, n = 2, n = 5, and n = 7, respectively) and % β‐globin protein (Figure 1E right: n = 2, n = 2, n = 6, and n = 7, respectively). n indicates the number of experiments, performed in triplicates. HPLC analysis was performed in single runs. BMP4, bone morphogenetic protein 4; EPO, erythropoietin; FBS, fetal bovine serum; FL, fms‐like tyrosine kinase 3 ligand; GPA, glycophorin A; IL3, interleukin‐3; KSR, knockout serum replacement; MEF, mouse embryonic fibroblasts; MT, Matrigel; qPCR, quantitative polymerase chain reaction; RP‐HPLC, reverse phase high pressure liquid chromatography; SCF, stem cell factor; TPO, thrombopoietin; VEGF, vascular endothelial growth factor

hES/iPSC‐derived erythroid cells were generated through ES/iPS‐sacs using a four‐step culture method, as previously described^1^, ^8^ (Figure 1A). Briefly, in the ES/iPS‐sac culture phase (step 1) clusters of hES/iPSCs (1 × 10^5^ cells per 100 mm dish from hESCs maintained on MEF or on MT) were cultured on irradiated C3H10T1/2 feeder cells (1.5e6 cells per 100 mm dish) for at least 15 days using Iscove's Modified Dulbecco's Medium (IMDM; Sigma Aldrich, Saint Louis, Missouri, https://www.sigmaaldrich.com/) supplemented with 1% ITS Liquid Media Supplement (Sigma Aldrich), 50 mg/mL ascorbic acid (Sigma Aldrich), 0.45 mM a‐monothioglycerol (Sigma Aldrich), 20 ng/mL human VEGF (PeproTech), 2 mM l‐glutamine, and 15% FBS or 20% knockout serum replacement (KSR, Thermo Fisher Scientific, http://www.thermofisher.com/). When we used hESCs maintained on MT (MT‐ES) and the media for ES‐sac generation containing KSR, the number of starting cells was reduced to 1 × 10^4^ to avoid the overcrowding of ES‐sacs, based on our preliminary experiments (data not shown).

Based on the hESCs maintenance and the media used for the ES‐sac generation, we included four groups: (a) hESCs maintained on MEF feeder cells using FBS‐based ES‐sac media (MEF‐FBS), (b) hESCs maintained on MEF feeder cells using KSR‐based ES‐sac media (MEF‐KSR), (c) hESCs maintained on Matrigel using FBS‐based ES‐sac media (MT‐FBS), and (d) hESCs maintained on Matrigel using KSR‐based ES‐sac media (MT‐KSR). The media was replaced every 2 or 3 days, and the ES/iPS‐sacs generated were harvested on days 15, 16, 17, 18, 19, or 20 of culture depending on the experiment. For the transfer phase (step 2), ES/iPS‐sacs were broken using a 15‐minute Collagenase IV incubation at 37°C followed by mechanical disruption using a 5‐mL pipet and filtration using a 40‐μm cell strainer. A fraction of the spherical cells was reserved for cell surface analysis, and the rest were cultured on irradiated OP9 feeder cells (ATCC) for 2 days using the same media used during the ES‐sac generation supplemented with 50 ng/mL fms‐like tyrosine kinase 3 ligand (FL; R&D systems, Minneapolis, Minnesota, https://www.rndsystems.com/), 50 ng/mL thrombopoietin (TPO; R&D systems), 5 ng/mL interleukin‐3 (IL3; R&D systems), 50 ng/mL stem cell factor (SCF; R&D systems), 5 U/mL erythropoietin (EPO; AMGEN, Thousand Oaks, California, http://www.amgen.com/), and 10 ng/mL bone morphogenetic protein 4 (R&D systems). In the erythroid expansion and differentiation phase (step 3), the suspension cells were collected and transferred into fresh culture plates including irradiated OP9 feeder cells (enhancing hemoglobinization), and cultured for 5 days in IMDM media containing 10 ng/mL SCF, 1.0 ng/mL IL3, 2.0 U/mL EPO, 1.0 μM estradiol (Pfizer, New York, http://www.pfizer.com/), 1.0 μM dexamethasone (VETone, Boise, Idaho, http://www.vetone.net/), and 20% FBS (or 20% KSR in iPS‐sac experiments). For the erythroid maturation phase (step 4), the media was substituted by IMDM containing 2% bovine serum albumin (Roche, Indianapolis, Indiana, http://www.roche.com/), 0.56 mg/mL transferrin (Sigma Aldrich), 2 mM l‐glutamine, 2.0 U/mL EPO, 10 ng/mL insulin (Lilly, Indianapolis, Indiana), and 20% FBS (or 20% KSR in iPS‐sac experiments), and the cells were cultured for another 8 to 10 days.

### 2.3 Gene correction of SCD‐iPSCs

**Figure 2:**
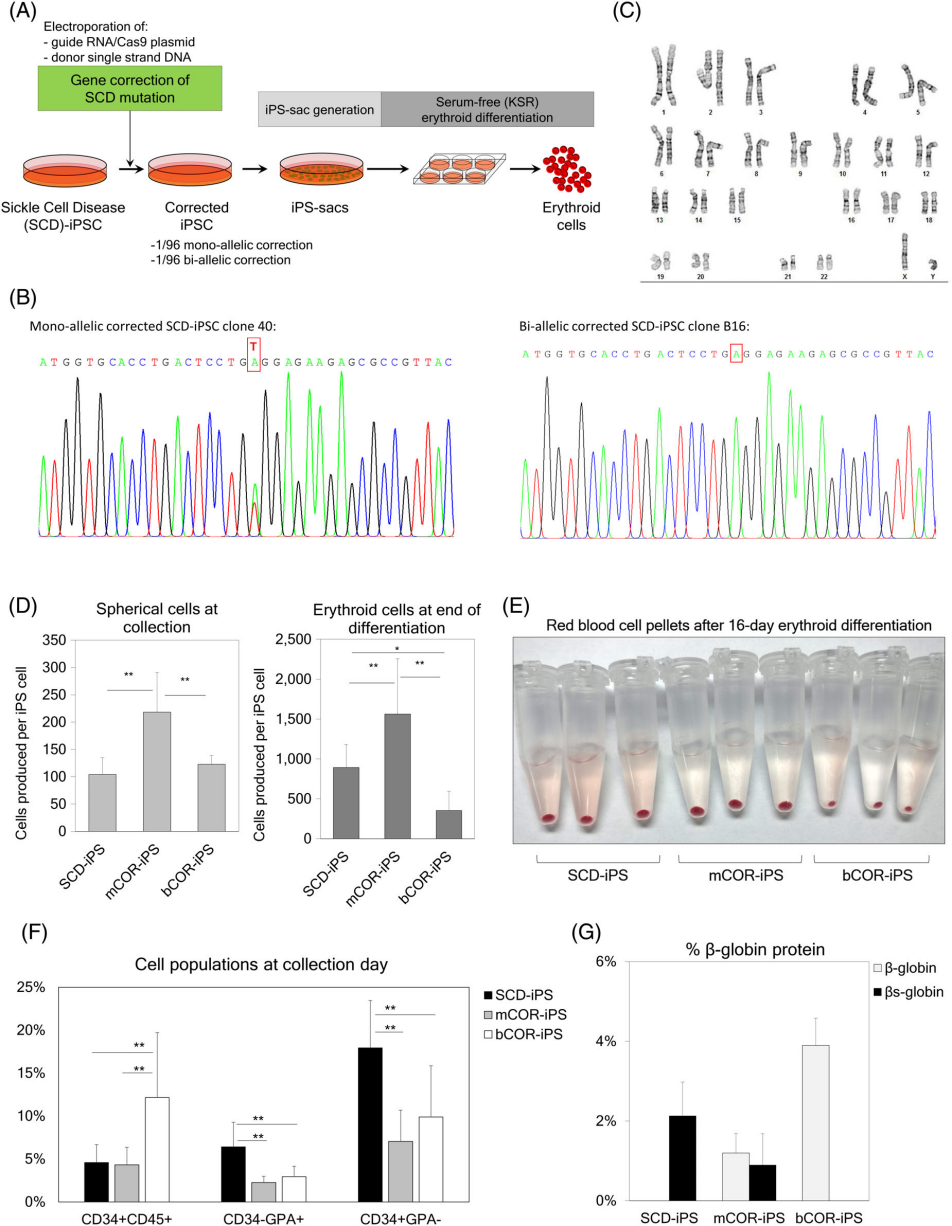
A, Gene correction of the sickle mutation in β‐globin gene in sickle cell disease (SCD)‐induced pluripotent stem cells (iPSCs), followed by our optimized serum‐free iPSC‐derived sac (iPS‐sac) generation and serum‐free erythroid differentiation. B, Nucleotide‐specific chromatogram peaks for monoallelic correction (mCOR, left panel) and biallelic correction (bCOR, right panel), analyzed by Sanger sequencing. C, Chromosomes with a Giemsa‐banding stain in a biallelic corrected iPSC line. D, Yields of iPS‐sac‐derived hematopoietic‐like spherical cells per iPSC at day 19 (left panel) and erythroid cells per iPSC after differentiation of the spherical cells (right panel). E, Red colored pellets of iPS‐sac‐derived erythroid cells from non‐corrected SCD iPSCs as well as gene‐corrected iPSCs (mCOR and bCOR). F, Percentages of cell surface markers in iPS‐sac‐derived spherical cells from SCD‐iPSCs, mCOR‐iPSCs, and bCOR‐iPSCs. G, Normal β‐globin and β^S^‐globin production at the protein level, analyzed by RP‐HPLC. Data reported as mean ± SD. Statistical analysis was performed by Tukey's honestly significant difference test (*P < .05 and **P < .01). SCD‐iPS, n = 4; mCOR‐iPS, n = 4; bCOR‐iPS, n = 4 for all the analysis

A guide RNA targeting the SCD mutation (5′‐GTA ACG GCA GAC TTC TCC AC‐3′) was inserted into a pCAG‐eCas9‐GFP‐U6‐gRNA plasmid (Addgene #79145) vector that expresses a guide RNA, a high‐fidelity version of Streptococcus pyogenes‐derived Cas9, and a green fluorescent protein (GFP) marker. A single‐strand oligodeoxynucleotide encoding a normal β‐globin sequence (5′‐GGC AGA GCC ATC TAT TGC TTA CAT TTG CTT CTG ACA CAA CTG TGT TCA CTA GCA ACC TCA AAC AGA CAC CAT GGT GCA CCT GAC TCC TGA GGA GAA GAG CGC CGT TAC TGC CCT GTG GGG CAA GGT GAA CGT GGA TGA‐3′) was synthesized by Integrated DNA Technologies, Inc (Skokie, Illinois) and included silent mutations (TCT to AGC, underlined) to introduce an HhaI enzyme site (GCGC) for restriction fragment length polymorphism (RFLP) screening of gene‐corrected clones. Two million SCD‐iPSCs were transfected with 6 μg CRISPR/Cas9 plasmid and 6 μg donor DNA using Amaxa's nucleofector 4D (Lonza, Basel, Switzerland) with a preset program for human H9 hESCs. Two days after electroporation, GFP‐positive cells were sorted, expanded, and plated into one cell/96‐well by serial dilution. The iPSC clones from 96‐well plates were subject to genomic DNA extraction and PCR amplification by primers (5′‐TGG TAT GGG GCC AAG AGA TA‐3′ and 5′‐CAG ATC CCC AAA GGA CTC AA‐3′), and RFLP screening by HhaI digestion. Gene‐corrected alleles were identified by the introduction of a HhaI site in the PCR products. The PCR products of the corrected SCD clones were further confirmed by Sanger sequencing (Figure 2B).

Karyotypes in iPSC lines were analyzed by Giemsa‐banding (WiCell) (Figure 2C) and KaryoStat (Thermo Fisher Scientific) (Figure S3A).

### 2.4 Flow cytometry

We performed cell surface analysis by incubating the cells with different antibodies depending on the analysis including CD31‐BV421 (clone WM59), CD34‐PE (clone 563), CD34‐FICT (clone 581), CD34‐PerCP Cy5.5 (clone 8G12), CD34‐BB700 (clone 8G12), CD36‐FICT (clone CB38), CD38‐APC H7 (clone HB7), CD41a‐FICT (clone HIP8), CD43‐APC (clone 1G10), CD45‐PE (clone HI30), CD45‐PE Cy7 (clone HI30), CD45RA‐Alexa Fluor 700 (clone HI100), CD49f‐PE CF594 (cloneGoH3), CD71‐APC (clone M‐A712), CD73‐BV605 (clone AD2), CD90‐PE (clone 5E10), CD144‐BUV395 (clone 55‐7H1), CD184‐BV786 (clone 12G5), GPA‐PE (CD235a clone GA‐R2), GPA‐PE Cy7 (CD235a clone GA‐R2), GPA‐BV650 (CD235a clone GA‐R2), and DLL4‐BV510 (clone MHD4‐46) (all from Becton Dickinson). Three different instruments were used depending on the experiment, including FACSCalibur (3‐colors analysis), BD Canto flow cytometer (6‐colors analysis), and BD Fortessa flow cytometer (14‐colors analysis) (the three instruments from Becton Dickinson, East Rutherford, New Jersey, http://www.bd.com/).

### 2.5 Reverse transcription quantitative polymerase chain reaction

After erythroid differentiation, erythroid cells were collected and evaluated to determine RNA expression levels of ε‐globin, γ‐globin, β‐globin, and α‐globin, as previously described.^17^ Total RNA was extracted from erythroid cells produced after differentiation using TRIZol LS (Life Technologies). Reverse transcription was performed using the SuperScript III kit (Life Technologies). Quantitative PCR assay was performed using gene‐specific primers and probes in the QuantStudio 6 Flex Real‐Time PCR System (ThermoFisher Scientific). The primer and probe sequences used^8^, ^17^, ^20^ (Integrated DNA Technologies, Inc, Coralville, Iowa) are listed in Table 1. We used a control plasmid containing one copy of ε‐, γ‐, β‐, and α‐globin cDNA and calculated relative amounts of ε‐, γ‐, and β‐globin RNA, which were standardized by α‐globin signals.

**Table 1:** Primers and probes for qPCR

Gene	Primer	Sequence
ε‐globin	Forward	5′‐ACA ACC TCA AGC CCG C‐3′
	Reverse	5′‐AGA CAC CAG CTT CTG CC‐3′
	Probe	5′‐HEX‐TGC CAA AGT GAG TAG CCA GAA TAA TC‐ZEN‐IBFQ‐3′
γ‐globin	Forward	5′‐ACC TGG ATG ATC TCA AGG G‐3′
	Reverse	5′‐CAG TCA CCA TCT TCT GCC‐3′
	Probe	5′‐Cy5‐TGC CGA AAT GGA TTG CCA AAA CGG TC‐IBRQ‐3′
β‐globin	Forward	5′‐ ACA ACC TCA AGG GCA CC‐3′
	Reverse	5′‐ACA CCA GCC ACC ACT TTC‐3′
	Probe	5′‐FAM‐ TGC CAA AGT GAT GGG CCA GCA CAC AG‐IBRQ‐3′
α‐globin	Forward	5′‐TCC CCA CCA CCA AGA CCT AC‐3′
	Reverse	5′‐CCT TAA CCT GGG CAG AGC C‐3′
	Probe	5′‐HEX‐TCC CGC ACT TCG ACC TGA GCC A‐IBRQ‐3

### 2.6 Reversed‐phase high‐performance liquid chromatography

We performed reversed‐phase high‐performance liquid chromatography (RP‐HPLC) for globin protein analysis, as previously described.^8^, ^20^, ^21^ Briefly, we collected ES/iPS‐sac‐derived erythroid cells, and after three washes with phosphate‐buffered saline (Corning, New York), the cells were lysed using HPLC grade water, vortexed, and mixed with 10% of 100 mM Tris (2‐carboxyethyl) phosphine (Thermo Fisher Scientific). After 5 minutes of incubation, the same volume of solution containing 0.1% trifluoroacetic acid (TFA) and 32% acetonitrile (Honeywell Burdick & Jackson, Morris Plains, New Jersey, https://labchemicals-honeywell.com/) was added to the lysed sample. After 16 000*g* of centrifugation for 5 minutes, the supernatant was injected and analyzed in 0.8 mL per minute flow rate for 50 minutes using the Agilent 1100 HPLC (Agilent Technologies) equipped with a reversed‐phase column, Aeris 3.6 lm Widepore C4 200 (25 034.6 mm, Phenomenex, Torrance, California, http://www.phenomenex.com/) with two solvents: solvent A, 0.12% TFA in water, and solvent B, 0.08% TFA in acetonitrile.

### 2.7 Statistical analysis

Statistical analysis was performed by the IBM SPSS Statistics version 1.0.0‐2482 (IBM Corp, Armonk, New York, http://www.ibm.com/DataStatistics/SPSS). All experiments were performed in triplicate. The difference between the two groups was evaluated by a two‐tailed *t*‐test. The difference between more than two groups was evaluated by one‐way analysis of variance using Dunnett's test when compared with a control group, or Tukey's honest significant difference test when compared among all the groups. A *P* value of <.05 or <.01 was deemed significant.

## 3 RESULTS

### 3.1 hESCs maintained on Matrigel and differentiated using a KSR‐based media improves ES‐sac and spherical cell generation with similar levels of β‐globin production after erythroid differentiation

Since feeder cell‐free iPSC maintenance is optimal for electroporation‐based delivery of gene correction tools, we evaluated feeder cell‐free culture for hESC maintenance followed by serum‐free ES‐sac generation. In hESC maintenance, mouse embryonic fibroblast (MEF) feeder cells were switched to Matrigel (MT) protein coating, and in ES‐sac generation, FBS was replaced by KSR.^22^ We investigated four different conditions: hESC maintenance on MEF followed by FBS‐based ES‐sac generation (MEF‐FBS, our standard),^8^, ^17^ hESC maintenance on MEF followed by KSR‐based ES‐sac generation (MEF‐KSR), hESC maintenance on Matrigel followed by FBS‐based ES‐sac generation (MT‐FBS), and hESC maintenance on Matrigel followed by serum‐free KSR‐based ES‐sac generation (MT‐KSR) (Figure 1A). KSR is composed of more defined materials than FBS, likely allowing for the reduction in variability among batches, as previously observed when using FBS.^23^, ^24^, ^25^ In preliminary ES‐sac generation analysis, feeder cell‐free hESC maintenance (with MT) as well as serum‐free ES‐sac protocol (with KSR) resulted in greater amounts of hematopoietic‐like spherical cells (*P* < .01), which was probably due to more efficient ES‐sac generation (*P* < .01) (Figure S1). In both conditions, ES‐sacs included slightly lower percentages of a CD34+CD45+ population (containing HSPC) (*P* < .05) and slightly lower percentages of a CD34−GPA+ population (producing a more primitive erythropoiesis producing ε‐globin, γ‐globin, and no β‐globin^17^) (*P* < .05), compared with our standard MEF‐FBS condition. We then compared all four conditions in parallel. ES‐sac generation in MT‐KSR resulted in 15‐fold greater amounts of spherical cells (*P* < .01) (Figure 1B, right panel) compared with the MEF‐FBS condition. A 2.2‐fold lower percentage of CD34+CD45+ HSPC populations, 2.0‐fold lower percentage of CD34‐GPA+ (*P* < .05), and similar percentage of CD34 + GPA− (more definitive hematopoiesis producing γ‐ and β‐globins without ε‐globin after erythroid differentiation^17^) were observed in MT‐KSR (not significant, ns) compared with MEF‐FBS (Figure 1D). These data demonstrate that the MT‐KSR condition is optimal for the production of greater amounts of ES‐sacs and hematopoietic‐like spherical cells, compared with our standard MEF‐FBS condition. Additionally, the MT‐KSR condition is preferable for clinical application, since the removal of FBS is an important step for xeno‐free culture.

To further characterize definitive erythropoiesis from the ES‐sacs among these four conditions, ES‐sac‐derived spherical cells were differentiated into erythroid cells, and globin production was measured at the RNA and protein levels. Up to 5.8‐fold greater amounts of erythroid cells were yielded from the MT‐KSR condition during erythroid differentiation (*P* < .05) compared with the MEF‐FBS condition (Figure 1C). In the two conditions for KSR‐based ES‐sac generation (MEF‐KSR and MT‐KSR), 4.1‐ to 4.6‐fold higher levels of ε‐globin RNA (*P* < .05) and 2.0‐ to 2.1‐fold lower levels of β‐globin RNA (*P* < .05) were detected compared with the MEF‐FBS condition (Figure 1E, first panel); however, there was no significant difference in β‐globin amounts at the protein level, as analyzed by RP‐HPLC (Figure 1E, second panel), suggesting that all four conditions result in definitive erythroid cells producing similar amounts of β‐globin. Taken together, MT‐KSR is the most optimal condition of the four groups for ES‐sac generation, since MT‐KSR‐based ES‐sac generation allowed for greater yields of ES‐sacs and hematopoietic‐like spherical cells with no reduction in β‐globin production at the protein level following erythroid differentiation.

**Figure 3:**
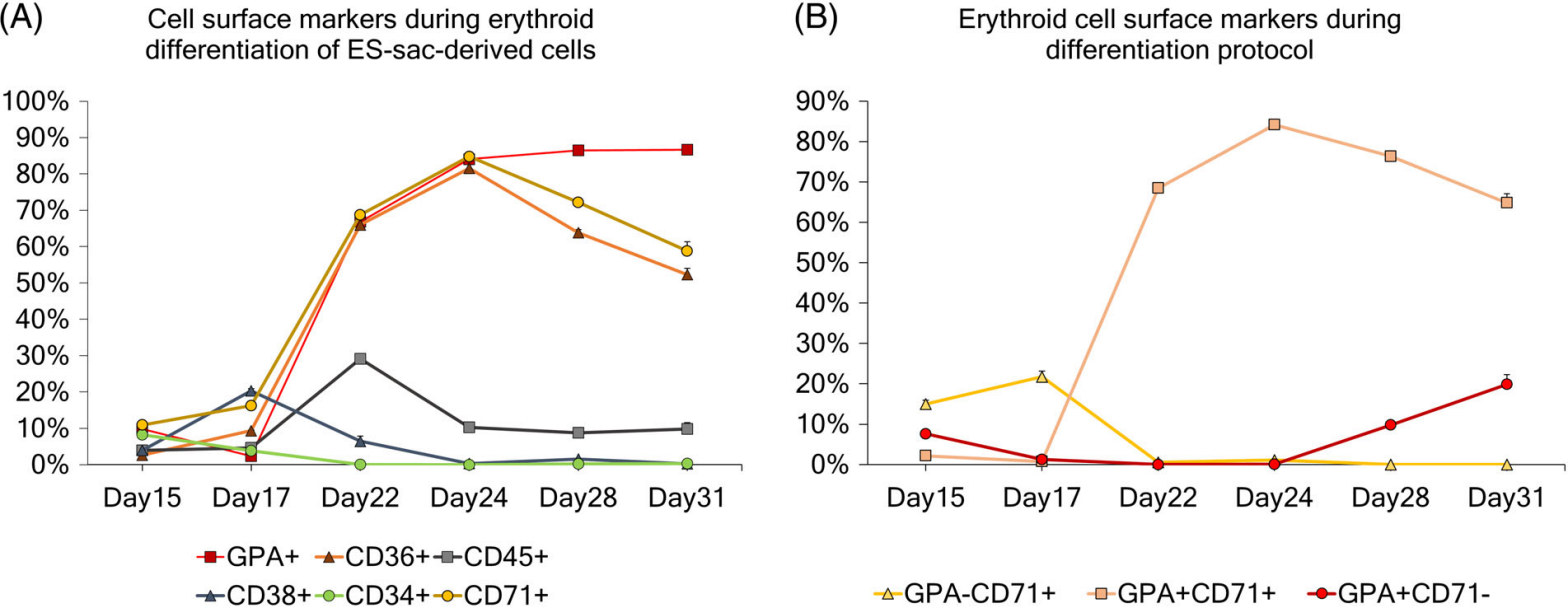
A, Evolution of different cell surface markers during serum‐free erythroid differentiation of MT‐KSR ES‐sac‐derived cells. B, Evolution of the different erythroid populations based on GPA and CD71 cell surface markers during the serum‐free erythroid differentiation protocol

We further performed detailed surface marker analysis during KSR‐based serum‐free erythroid differentiation from 15‐day MT‐KSR ES‐sacs at various time points (day 15‐31) (Figure 3A). Expectedly, a hematopoietic progenitor marker of CD34 expression decreased from the beginning of differentiation to undetectable levels on day 22. Nonspecific hematopoietic markers CD38 and CD45 peaked on day 17 and day 22, respectively, and decreased to lower levels during erythroid maturation. In contrast, all erythroid markers including CD36, CD71, and GPA increased drastically on day 17‐24. Both CD36 and CD71 peaked on day 24 and decreased to the end of differentiation (day 31), whereas GPA gradually increased and achieved up to ~90%, demonstrating that most cultured cells are differentiated into the erythroid lineage at the end of differentiation. In the dual‐color analysis of CD71 and GPA, GPA+CD71+ immature erythroid cells rapidly increased on day 17‐24, and then GPA+CD71− mature erythroid cells emerged on day 28‐31, reflecting the maturation of erythroid cells (Figures 3B and S4).^26^, ^27^

### 3.2 Extension of ES‐sac generation culture results in greater amounts of hematopoietic‐like spherical cell generation and higher percentages of the CD34+CD45+ HSPC population

We previously demonstrated that the longer duration of ES‐sac generation (more matured ES‐sacs) increases the CD34+CD45+ HSPC and CD34+GPA− definitive cell populations, resulting in higher amounts of β‐globin production after erythroid differentiation.^17^ However, when culture media contains FBS, longer duration of ES‐sac generation (up to 15‐17 days) results in the breakage of ES‐sac structures, release of hematopoietic‐like spherical cells from broken ES‐sacs, and difficult collection and analysis of these cells. Since KSR‐based ES‐sac media produces more compact ES‐sacs, we hypothesized that KSR‐based media would allow us to extend the duration (longer than 17 days) of ES‐sac generation without breakage of ES‐sac structure. We cultured hESCs in ES‐sac generation media for 15 (standard duration), 16, 17, 18, 19, and 20 days, and evaluated the yields of both ES‐sac‐derived spherical cells and differentiated erythroid cells as well as the cell populations contained in the spherical cells.

**Figure 4:**
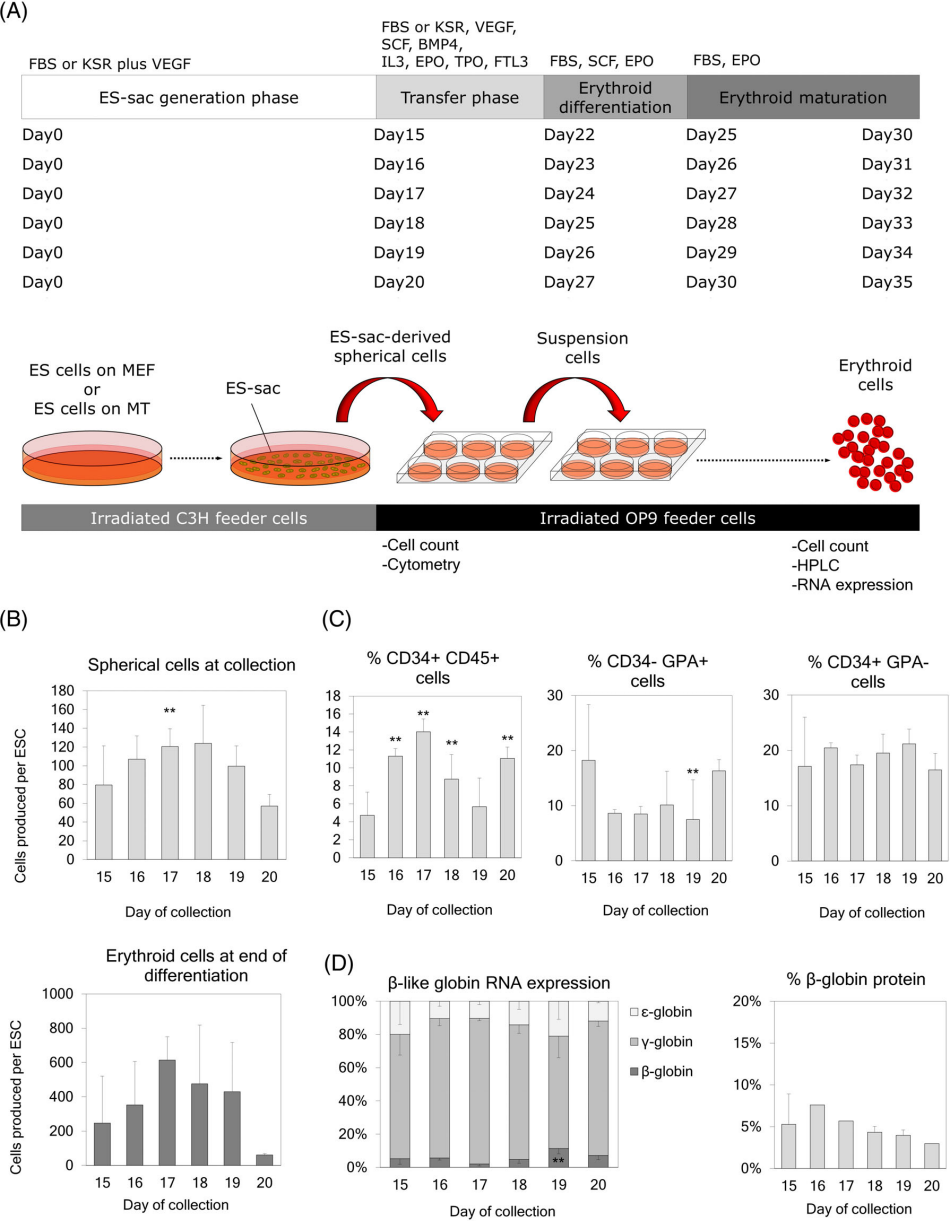
A, Timeline for extension of ES‐sac generation culture (15, 16, 17, 18, 19, and 20 days). B, Upper panel: Yield of ES‐sac‐derived spherical cells per hESC for various durations of ES‐sac generation. Lower panel: Yield of erythroid cells produced per hESC from spherical cells from different collection times. C, Percentages of cell surface markers in ES‐sac‐derived spherical cells after various durations of ES‐sac generation. D, Left panel: β‐like globin gene RNA expression after erythroid differentiation. Right panel: β‐globin production at the protein level, analyzed by RP‐HPLC. Data reported as mean ± SD. Statistical analysis was performed by Dunnett's test, compared with the 15‐day collection group (**P < .01). Day 15, n = 15; Day 16, n = 1; Day 17, n = 1; Day 18, n = 3; Day 19, n = 5; Day 20, n = 1 for all the analysis except for RNA analysis (Figure 4D left: n = 7, n = 1, n = 1, n = 3, n = 2, and n = 1, respectively) and β‐globin protein analysis (Figure 4D right: n = 7, n = 1, n = 1, n = 3, n = 2, and n = 1, respectively). n indicates the number of experiments, performed in triplicates. HPLC analyses were performed in single runs. hESC, human embryonic stem cell; RP‐HPLC, reverse phase high pressure liquid chromatography

Our optimized MT‐KSR condition allowed for the maintenance of non‐disrupted ES‐sac structures up to 20 days (Figure S2). Extension of the ES‐sac generation increased spherical cell yields, reaching up to 1.6‐fold greater amounts of cells from 18‐day ES‐sac generation (*P* < .01) compared with 15 days. After longer than 18 days of ES‐sac culture, the number of spherical cells decreased to similar levels as that observed at day 15 (Figure 4B, upper panel). A similar trend was observed in erythroid cell yields among different spherical‐cell‐collection days, reaching up to 2.5‐fold greater numbers of erythroid cells from 17‐day ES‐sac generation (ns) compared with 15 days (Figure 4B, bottom panel). Extended duration of ES‐sac generation resulted in up to a 3.0‐fold greater percentage of the CD34+CD45+ HSPC population compared with our standard duration of 15 days (*P* < .01 except day 19) (Figure 4C, first panel). The GPA+CD34− primitive cell population was up to 2.4‐fold lower in the extended conditions compared with the 15‐day condition (*P* < .01 on day 19 only) (Figure 4C, second panel), whereas similar levels of the GPA−CD34+ definitive cell population were observed (ns) among all the conditions (Figure 2C, third panel). These data demonstrate that KSR‐based serum‐free media allows for the extension of ES‐sac generation without disruption and extended ES‐sac generation results in greater amounts of spherical cells and CD34+CD45+ HSPC generation, reaching similar percentages of CD34+CD45+ HSPC population in spherical cells compared with our standard MEF‐FBS condition.

**Figure 5:**
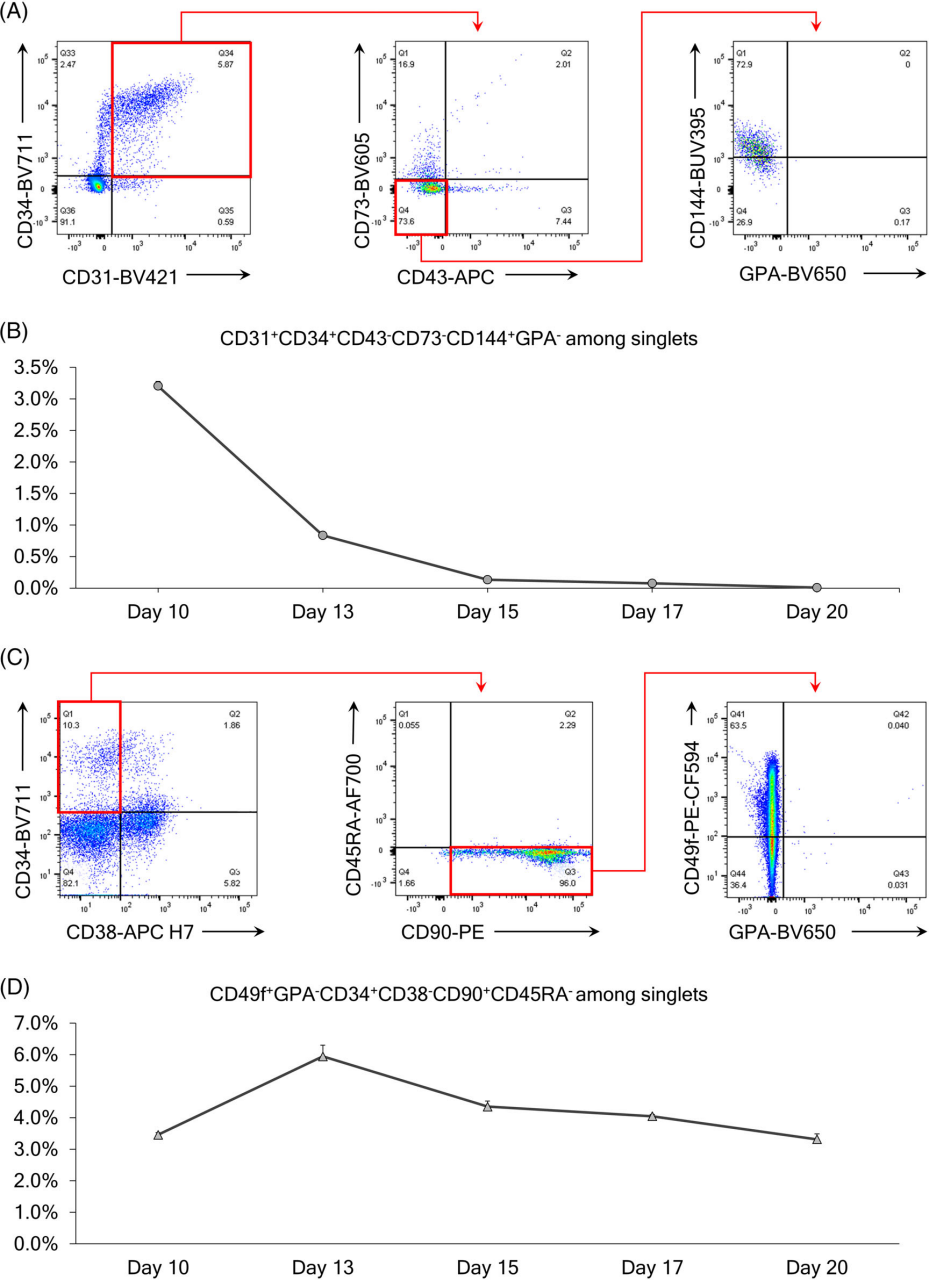
A, Flow cytometry selection panel for hemogenic endothelium cells from singlets. B, Evolution of hemogenic endothelium during the MT‐KSR ES‐sac generation protocol. C, Flow cytometry selection panel for HSPCs from singlets. D, Evolution of HSPCs during the MT‐KSR ES‐sac generation protocol. HSPC, hematopoietic stem/progenitor cell; KSR, knockout serum replacement; MT‐KSR, Matrigel using KSR‐based ES‐sac media

To further investigate emergence of cell populations within MT‐KSR ES‐sacs, we evaluated a CD34+CD31+CD43−CD73−CD144+GPA− hemogenic endothelium (HE) cell population^8^, ^28^, ^29^, ^30^ as well as a CD34+CD38−CD90+CD45RA−CD49f+more precise HSPC population^31^, ^32^ at various time points (day 10‐20).^18^, ^33^, ^34^ A CD34+CD31+CD43−CD73−CD144+GPA− HE population was detected on day 10 and decreased to undetectable levels day 20 after ES‐sac generation (Figure 5A). In contrast, a CD34+CD38−CD90+CD45RA−CD49f+HSPC population peaked on day 13 and remained at the all time points analyzed (Figure 5B). These data suggest that the HSPC population was generated following HE emergence, and all time points of ES‐sac‐derived spherical cells could be differentiated to definitive erythroid cells through HSPCs.

After erythroid differentiation, the RNA expression of all the β‐globin series was similar among all extended conditions and the standard 15‐day condition, except for a 2.2‐fold higher β‐globin RNA expression in erythroid cells differentiated from spherical cells collected at day 19 of ES‐sac generation compared with that of day 15 (*P* < .05) (Figure 4D, first panel). Similar levels of β‐globin production were detected at the protein level by RP‐HPLC in all conditions. (Figure 4D, second panel). Overall, these data suggest that around the 18‐day (17‐19 days) ES‐sac generation on MT‐KSR condition is optimal for greater amounts of hematopoietic‐like spherical cells and CD34+CD45+ HSPCs with similar amounts of β‐globin production at the protein level after erythroid differentiation.

### 3.3 Gene correction of the SCD mutation in the β‐globin gene in SCD iPSCs confirmed at DNA and protein levels

To investigate a regenerative medicine strategy for SCD, we performed gene correction of the SCD mutation in the β‐globin gene on an iPSC line generated from SCD patient bone marrow stromal cells^8^ followed by iPS‐sac generation and erythroid differentiation (Figure 2A). To perform gene correction in SCD iPSCs, Matrigel condition was used for iPSC maintenance, since a feeder cell‐free condition is optimal for the delivery of CRISPR/Cas9 tools with electroporation. Two million SCD iPSCs were used for electroporation to deliver a single‐stranded donor DNA encoding the normal β‐globin sequence, a plasmid encoding both high‐fidelity Cas9 and a guide RNA targeting the SCD mutation site, and a GFP marker gene. After electroporation, we sorted GFP‐positive cells by flow cytometry, expanded single clones, and performed restriction enzyme‐based genetic screening. We found two gene‐corrected clones (2/96) with one monoallelic gene correction (mCOR‐iPS; Figure 2B, left panel) mimicking the SCD trait (normal β‐globin for one allele and β^s^‐globin for the other allele), and one biallelic gene‐corrected clone (normal β‐globin for both alleles) (bCOR‐iPS; Figure 2B, right panel). We confirmed a normal karyotype in both gene‐corrected iPSCs by chromosome staining (Giemsa banding) (Figure 2C) as well as array‐based comparative genomic hybridization (KaryoStat) (Figure S3a).

Using the optimized MT‐KSR condition, we generated iPS‐sacs from non‐corrected SCD iPSCs (without gene correction), mCOR‐iPSCs, and bCOR‐iPSCs. The iPS‐sac‐derived spherical cells were differentiated into erythroid cells using KSR‐based serum‐free erythroid differentiation media.^21^ Greater amounts of spherical cells (after iPS‐sac generation) and erythroid cells (after erythroid differentiation from spherical cells) were produced from the mCOR‐iPSCs (*P* < .01), which could potentially be due to the genetic variance among clones (Figure 2D). We also observed variance in the type of cell populations produced by the clones: higher percentages of CD34+CD45+ HSPCs in the bCOR‐iPSC‐derived spherical cells (*P* < .01), and higher percentages of both CD34−GPA+ primitive and CD34+GPA− definitive cell populations in iPS‐sacs (*P* < .01) (Figure 2F). However, after erythroid differentiation from iPS‐sac‐derived spherical cells, we obtained red colored pellets from all three iPSC clones, demonstrating robust hemoglobinization (Figure 2E). We evaluated β‐globin production at the protein level by RP‐HPLC, which demonstrated only β^S^‐globin in SCD‐iPSC‐derived erythroid cells, both β^S^‐globin and normal β‐globin in mCOR‐iPSC‐derived erythroid cells, and only normal β‐globin in bCOR‐iPSC‐derived erythroid cells (Figure 2G and Figure S3B). These data demonstrate that our serum‐free iPS‐sac protocol results in definitive erythroid cell generation with β‐globin protein production, allowing for proper evaluation of the SCD mutation correction in the β‐globin gene at both the DNA and protein levels. Thus, gene‐corrected iPSC technology may be applicable for the development of regenerative medicine for SCD.

## 4 DISCUSSION

We demonstrated viral vector‐free biallelic β‐globin gene correction in SCD iPSCs, which was confirmed by DNA sequencing and protein analysis by RP‐HPLC. To do this, we performed extensive optimization of the ES‐sac generation protocol including four different conditions to develop feeder cell‐free ES/iPS cell maintenance as well as serum‐free ES/iPS‐sac culture system. The serum‐free ES/iPS‐sac protocol resulted in the robust generation of ES/iPS‐derived RBCs producing normal β‐globin protein. This serum‐free ES/iPS‐sac protocol can help to reduce the variability caused by differences between FBS lots^23^, ^24^, ^25^ and reduce the risk of using animal‐derived products in a transplantation setting.^35^, ^36^ When hESC maintenance methods were changed to a feeder cell‐free culture suitable for genome editing, hESCs maintained on Matrigel (MT‐ES) slightly improved the efficiency of ES‐sac generation, resulting in greater amounts of hematopoietic‐like spherical cells. In addition, our serum‐free ES‐sac media increased the yields of ES‐sacs, hematopoietic‐like spherical cells, and spherical cell‐derived erythroid cells compared with the FBS‐containing media. This serum‐free protocol allowed for the lengthening of the ES‐sac generation duration, resulting in greater CD34+CD45+ HPSC population. Therefore, our serum‐free ES/iPS‐sac protocol with gene correction is potentially useful for developing a clinically applicable regenerative medicine strategy for SCD.

We demonstrated that either Matrigel‐based feeder cell‐free hESC maintenance (MT‐ES) or KSR‐based serum‐free ES‐sac media (ES‐KSR) allows for a more efficient ES‐sac generation, with greater amounts of hematopoietic‐like spherical cells compared with the MEF feeder cell‐based hESC maintenance (MEF‐ES) or FBS‐containing ES‐sac media (ES‐FBS), respectively. A significant difference in ES‐sac generation efficiency and ES‐sac morphology was observed between MT‐ES and MEF‐ES, even though similar pluripotent functions have been described for these ES‐sac maintenance conditions.^37^ The more efficient ES‐sac generation in the MT‐ES condition could have been caused by the binding of ES‐sacs to matrix proteins on the Matrigel‐coated culture dishes, which could have potentially induced activation of Wnt/β‐catenin signaling or other pathways.^38^ After several verifications, we concluded that it mainly changes the shape of ES‐sacs; however, the ES‐sac numbers per dish are slightly increased when using ES cells cultured in feeder‐free condition (MT‐ES), producing similar amounts of hematopoietic progenitor cells and erythroid cells. These results demonstrate that both feeder‐on (MEF‐ES) and feeder‐free (MT‐ES) ESCs produce similar qualities of ES‐sacs. We also observed emergence of CD34+CD31+CD43−CD73−CD144+GPA− HE population on day 10 during ES‐sac generation that might explain the day‐13 peak of CD34+CD38−CD90+CD45RA−CD49f+ HSPC population. In addition, continuous serum‐free hESCs culture from hESC maintenance to ES‐sac generation (as well as erythroid differentiation) might allow for more efficient cell growth, since hESCs did not exhibit a need to adapt from the serum‐free culture condition to the FBS‐containing culture condition. Recently, we developed a KSR‐based serum‐free erythroid differentiation protocol using primary human CD34+ HSPCs,^21^ which was adapted and implemented for erythroid differentiation in the latter part of this ES/iPSC research, possibly reducing the variability of FBS lots, and further allowed for similar amounts of ES/iPSC‐derived erythroid cell generation and β‐globin production at the protein level (Figure S3d). In addition, OP9 feeder cells were used for serum‐free erythroid differentiation, since the use of OP9 feeder cells enhanced hemoglobinization of erythroid cells differentiated from ES‐sac‐derived spherical cells with a similar ratio of β‐globin protein production, compared with feeder cell‐free erythroid differentiation (data not shown), allowing for more precise globin protein analysis in RP‐HPLC.

Between the four different ES/iPSC culture combinations (MEF‐FBS, MEF‐KSR, MT‐FBS, and MT‐KSR), MT‐KSR produced the greatest amounts of ES‐sacs, spherical cells, and erythroid cells, but slightly lower percentages of the CD34+CD45+ HSPC population in ES‐sac‐derived spherical cells compared with our standard MEF‐FBS condition. However, similar levels of β‐globin production were measured by RP‐HPLC among all four conditions, which was consistent with the similar percentages of the CD34+GPA− definitive population observed.^17^ To improve the CD34+CD45+ HSPC generation in MT‐KSR‐based ES‐sacs, we optimized the harvest timing of ES‐sac‐derived spherical cells by lengthening ES‐sac generation culture duration, which resulted in greater yields of hematopoietic‐like spherical cells and erythroid cells as well as higher percentages of the CD34+CD45+ HSPC population, similar to that achieved with our standard MEF‐FBS‐derived ES‐sac generation duration of 15 days. These data suggest that KSR‐based serum‐free ES‐sac generation results in slower differentiation to hematopoietic cells in ES‐sacs, compared with the FBS‐containing media. β‐globin production is similar at the protein level for all time points, consistent with the similar percentages of the CD34+GPA− definitive population observed.^17^

Using a method previously developed by our group, we performed viral vector‐free CRISPR/Cas9‐based gene correction of the SCD mutation in a SCD iPSC line (derived from SCD patient bone marrow stromal cells) that was maintained using our optimized MT‐ES culture condition.^8^ Our restriction enzyme‐based screening system for iPSC genome editing identified 2 clones of gene‐corrected iPSC lines from a screening of 96 clones. The gene correction efficiency in human iPSCs was strongly improved by these methods compared with our previous publication^11^; however, this efficiency is much lower than gene correction in human CD34+ HSPCs.^39^ Unlike HSPCs, corrected iPSCs can be isolated by cloning steps even at lower efficiencies of gene correction. Using our optimized serum‐free ES‐sac generation followed by serum‐free erythroid differentiation, gene‐corrected iPSCs can efficiently generate ES‐sacs, ES‐sac‐derived spherical cells, and spherical cell‐derived erythroid cells, which demonstrate similar amounts of normal β‐globin production at the protein level compared with the β^S^‐globin production from non‐corrected SCD iPSCs. Interestingly, both normal β‐globin and β^S^‐globin peaks were detected in RP‐HPLC from monoallelic corrected iPSCs. Recently, another group also reported a biallelic gene correction at the RNA level in SCD iPSCs using guide RNA/Cas9 RNP electroporation and AAV6 donor vector transduction.^15^ However, massive DNA synthesis from AAV vectors could result in various random integrations of rearranged vector sequences in target cells.^40^ Here, we report viral vector‐free biallelic correction and quantitative data of corrected β‐globin protein production in erythroid cells differentiated from gene‐corrected human SCD iPSCs.^11^, ^15^, ^41^, ^42^

## 5 SUMMARY

In summary, we demonstrate that compared with the FBS‐based media, our serum‐free culture media improves the yield of ES/iPS‐sacs containing CD34+CD45+ HSPCs, allowing for a greater production of erythroid cells expressing β‐globin protein. Furthermore, we performed viral vector‐free gene correction of the SCD mutation in an SCD iPSC line using this optimized serum‐free ES/iPS‐sac culture, and the biallelic corrected iPSCs generated iPS‐sac‐derived erythroid cells with normal β‐globin production, which was confirmed at DNA and protein levels. Our serum‐free ES/iPS‐sac protocol has the potential to contribute to the development of a xeno‐free strategy for iPSC‐based regenerative medicine, which is needed for the safe and reliable clinical application.

## CONFLICT OF INTEREST

The authors declared no potential conflicts of interest.

## AUTHOR CONTRIBUTIONS

J.J.H.M.: conception and design, collection and assembly of data, data analysis and interpretation, manuscript writing; N.U.: conception and design, data analysis and interpretation, manuscript writing; S.D.: conception and design, collection of data; Q.W.: collection of data; J.Z.: conception and design; J.T.: conception and design, manuscript writing, financial support, final approval of the manuscript.

## Supporting information


**Appendix** **S1.** Supporting Information.

## Data Availability

All relevant data are available from the corresponding author upon reasonable request.
