# Supplementary material for: Biallelic correction of sickle cell disease‐derived induced pluripotent stem cells (iPSCs) confirmed at the protein level through serum‐free iPS‐sac/erythroid differentiation
Source: Stem Cells Transl Med. 2020 Feb 7;9(5):590–602. doi: 10.1002/sctm.19-0216 (PMC7180291; doi:10.1002/sctm.19-0216)
Supplement: Supplementary file 1 — Appendix S1. Supporting Information. [file SCT3-9-590-s001.pdf]

SUPPLEMENTARY INFORMATION

Feeder cell-free hESC maintenance followed by serum-free ES-sac generation improved the yields of hematopoietic-like spherical cells as well as β-globin-producing erythroid cells

Since feeder cell-free iPSC maintenance is optimal for electroporation-based delivery of gene correction tools, we investigated how feeder cell-free culture for hESC maintenance affects ES-sac generation by using (1) hESCs maintained on mouse embryonic fibroblasts (MEF-ES) and (2) hESCs maintained on Matrigel Matrix coating (MT-ES) (Figure S1a). ES-sacs were generated from both MEF-ES and MT-ES using ES-sac media containing VEGF, and 15 days later, MT-ES produced 26-fold greater numbers of ES-sacs ($p<0.01$) and 11-fold greater amounts of hematopoietic-like spherical cells per hESC ($p<0.01$) as compared to MEF-ES (Figure S1b). Among ES-sac-derived spherical cells, the percentages of the CD34+CD45+ population were 1.4-fold lower in ES-sacs generated from MT-ES, compared to MEF-ES-derived ES-sacs ($p<0.05$) (Figure S1c). The CD34-GPA+, more primitive population, was 1.6-fold lower in ES-sacs produced from MT-ES, compared to MEF-ES ($p<0.01$) (Figure S1c). The percentages of CD34+GPA-, related to a more definitive cell population, were similar for both groups (ns) (Figure S1c). These data demonstrate that ES-sac generation from MT-ES results in greater amounts of ES-sac-derived spherical cells and different cell populations produced by those cells as compared to MEF-ES, even though it has been demonstrated that MEF-ES and MT-ES behave similarly for pluripotency [1].

We then developed an ES-sac generation protocol in serum-free media by replacing FBS with KSR. ES-sac generation in ES-KSR resulted in a 21-fold increase of ES-sac numbers and 2.8-fold greater amounts of spherical cells as compared to ES-sac generation with FBS-containing

media (ES-FBS) ($p<0.01$) (Figure S1d). 1.5-fold lower percentages of the CD34+CD45+ HSPC population ($p<0.01$), 1.3-fold lower percentages of the CD34-GPA+ primitive cell population ($p<0.05$), and similar percentages of the CD34+GPA- definitive cell population (ns) were observed as compared to ES-FBS (Figure S1e). These data demonstrate that KSR-based ES-sac generation (ES-KSR) increased efficiency of ES-sac generation and hematopoietic-like spherical cell production.

SUPPLEMENTARY FIGURES

Figure S1. (a) ES-sac generation from hESCs maintained on MEF feeder cells (MEF-ES) or Matrigel coating (MT-ES). **(b)** Upper panel: yield of ES-sacs per culture dish after 15-day culture. Lower panel: yield of ES-sac-derived spherical cells per hESC at day of collection (day 15). **(c)** Percentages of different cell populations in ES-sac-derived spherical cells determined by cell cytometry in MT-ES compared to MEF-ES. **(d)** Upper panel: yield of ES-sacs per culture dish after 15-day culture. Lower panel: yields of ES-sac-derived spherical cells per hESC at collection day (day 15). **(e)** Percentages of different cell populations in ES-sac-derived spherical cells determined by cell cytometry in FBS-ES compared to KSR-ES. Data reported as mean ± standard deviation. Statistical analysis was performed by two-tailed t-test (*$p<0.05$ and **$p<0.01$). MEF-ES n=6; MT-ES n=26 and ES-FBS n=14; KSR n=18. The n indicates number of experiments, performed in triplicates.

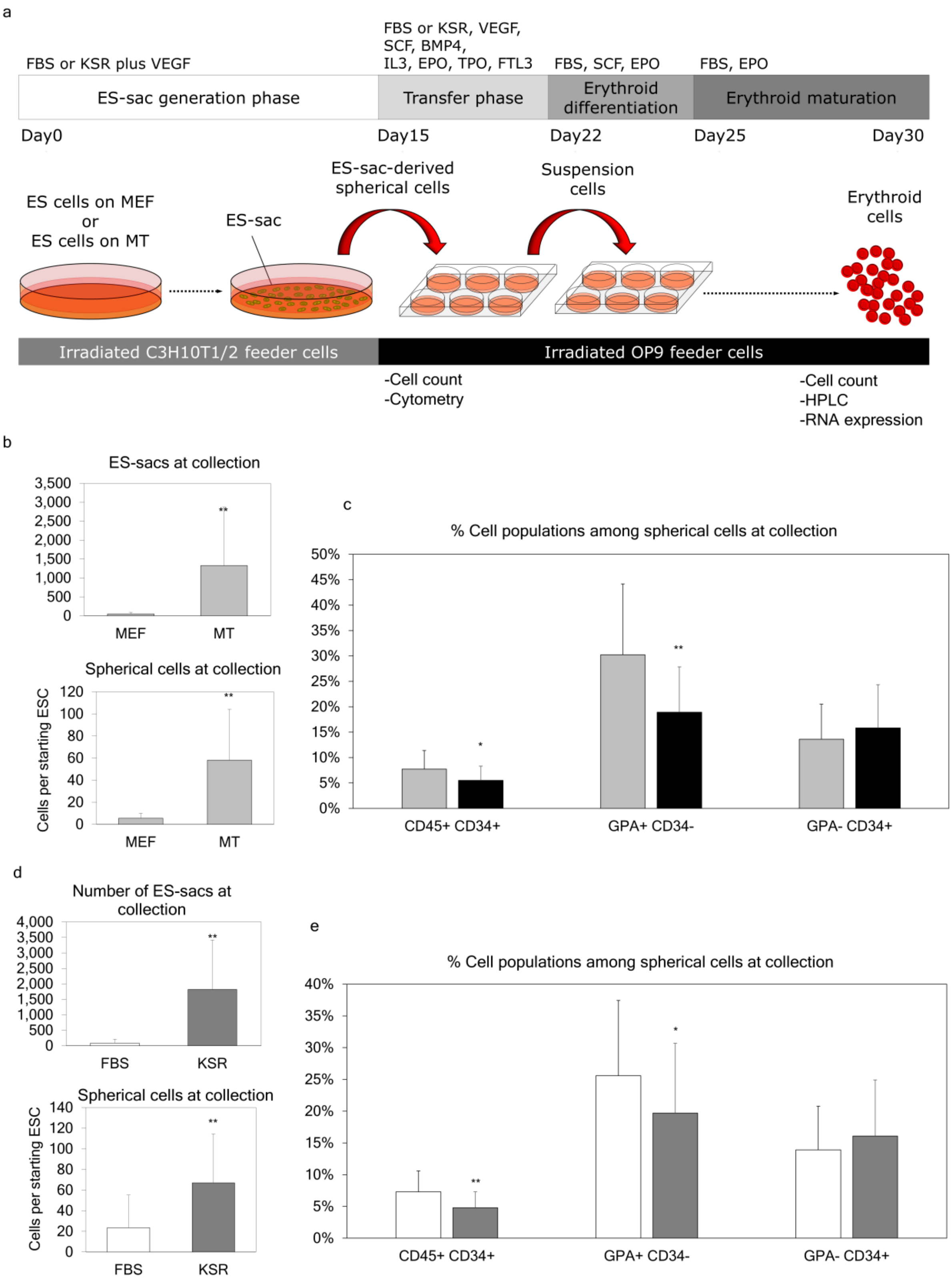
a
FBS or KSR plus VEGF
FBS or KSR, VEGF, SCF, BMP4, IL3, EPO, TPO, FTL3
FBS, SCF, EPO
FBS, EPO
ES-sac generation phase
Transfer phase
Erythroid differentiation
Erythroid maturation
Day0
Day15
Day22
Day25
Day30
ES cells on MEF or ES cells on MT
ES-sac
ES-sac-derived spherical cells
Suspension cells
Erythroid cells
Irradiated C3H10T1/2 feeder cells
Irradiated OP9 feeder cells
-Cell count
-Cytometry
-Cell count
-HPLC
-RNA expression
b
ES-sacs at collection
MEF
MT
Spherical cells at collection
Cells per starting ESC
c
% Cell populations among spherical cells at collection
CD45+ CD34+
GPA+ CD34-
GPA- CD34+
d
Number of ES-sacs at collection
FBS
KSR
Spherical cells at collection
Cells per starting ESC
e
% Cell populations among spherical cells at collection
CD45+ CD34+
GPA+ CD34-
GPA- CD34+

Figure S2. (a) ES-sac maintained for 15 days (100X magnification). **(b)** ES-sac maintained for 20 days (100X magnification).

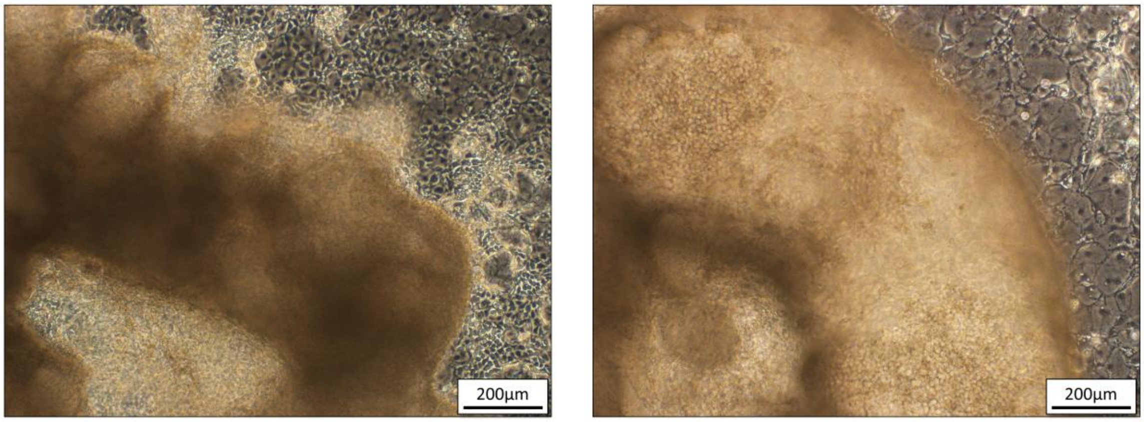

Figure S3. (a) Karyostat results showing copy number vs chromosome number. Peaks represent regions of amplification compared to reference genome. **(b)** HPLC histograms represent globin peaks with specific retention times used to determine amounts of β- and β^S-globin protein in iPS-sac-derived erythroid cells from SCD-iPSCs (upper graph), mCOR-iPSCs (lower left graph), and bCOR-iPSCs (lower right graph). **(c)** Total amounts of β- and β^S-globin protein in iPS-sac-derived erythroid cells. **(d)** Left: number of erythroid cells produced from hESCs using serum-free ES-sac media and either FBS- or KSR-based erythroid differentiation media during erythroid differentiation protocol; Right: amounts of β-globin protein determined by RP-HPLC contained by erythroid cells after differentiation protocol using either FBS- or KSR-differentiation media. Data reported as mean ± standard deviation. Statistical analysis was performed by Tukey's honestly significant difference test (*p<0.05 and **p<0.01). SCD-iPS n=4; mCOR-iPS n=4; bCOR-iPS n=3. The n indicates number of experiments, performed in triplicates.

a

1. KaryoStat™ analysis of CUB12096-7 revealed the sample originated from a male individual.
2. No chromosomal aberrations were found when comparing against the reference dataset

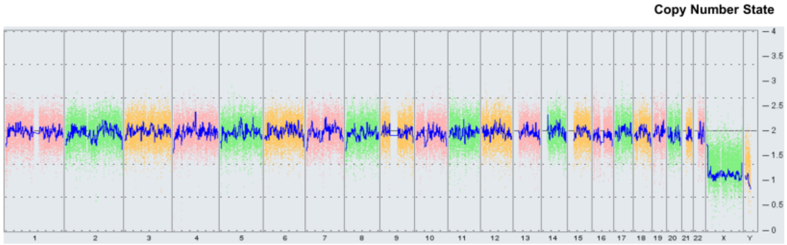

b

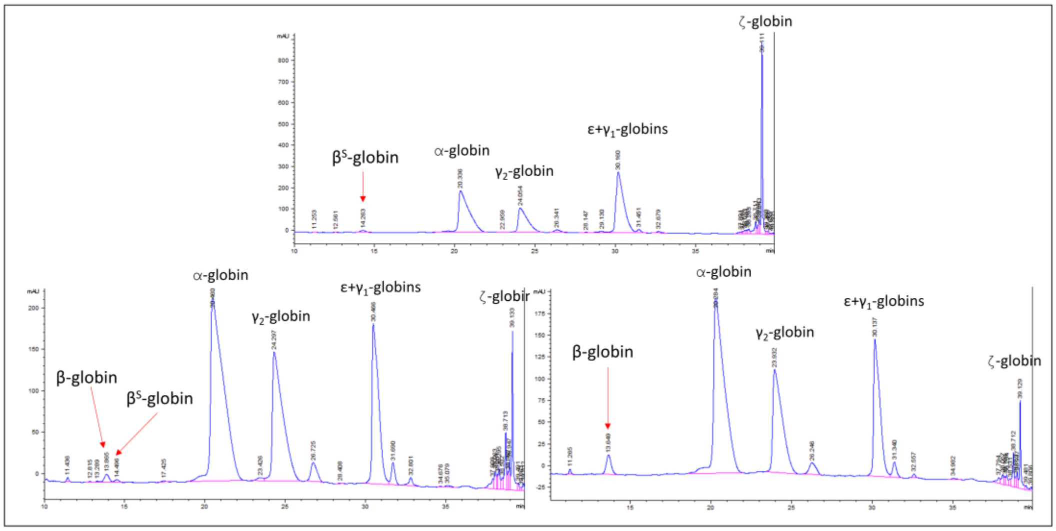

c

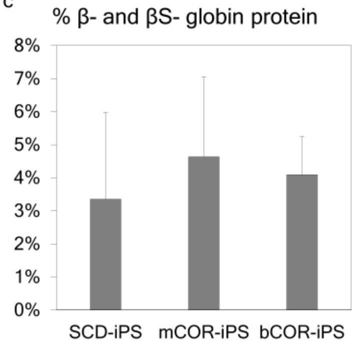

d

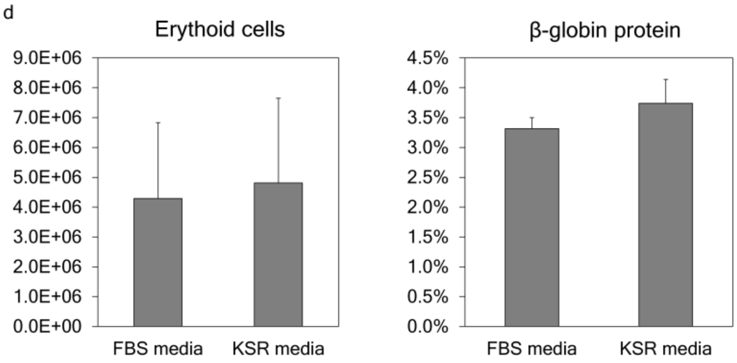

Figure S4. Evolution of GPA and CD71 cell surface markers during serum-free erythroid differentiation protocol of MT-KSR ES-sac-derived cells.

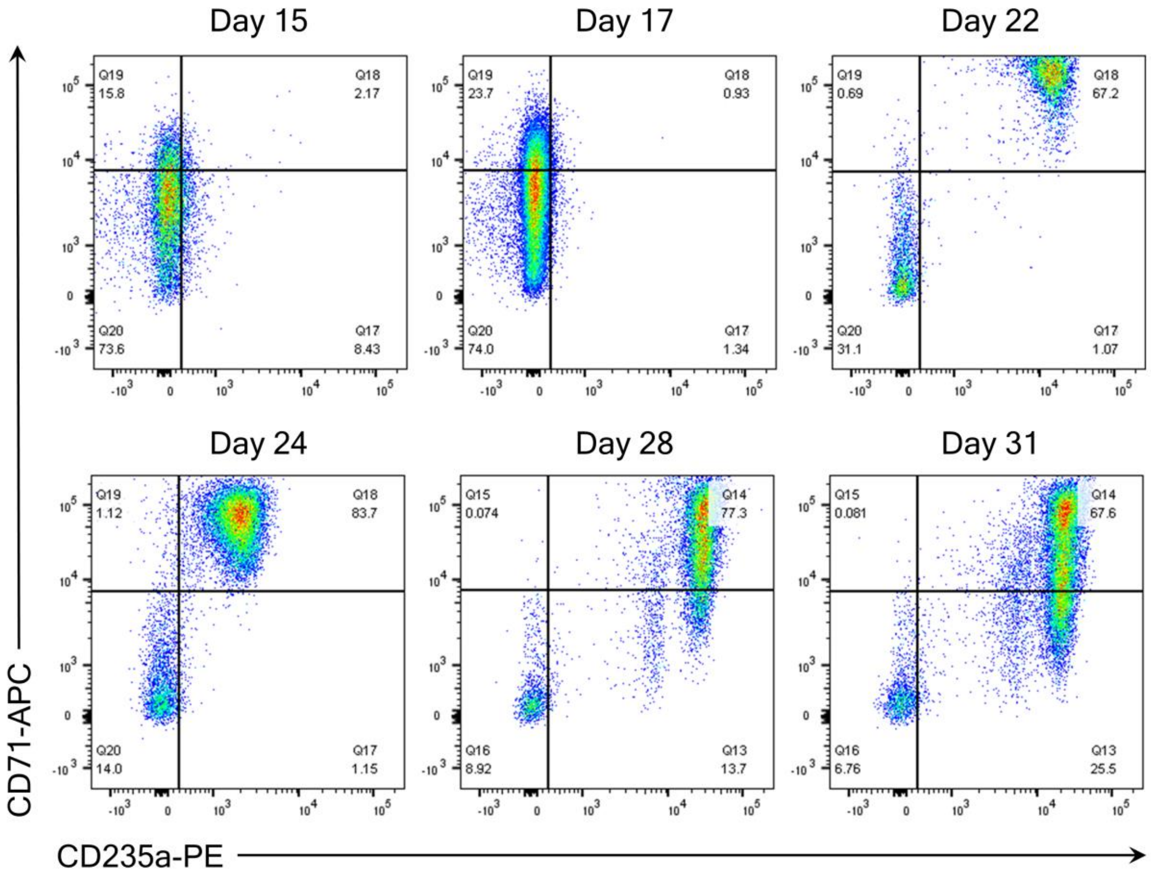

References

1. Ghasemi-Dehkordi, P., et al., *Comparison between the cultures of human induced pluripotent stem cells (hiPSCs) on feeder-and serum-free system (Matrigel matrix), MEF and HDF feeder cell lines.* J Cell Commun Signal, 2015. **9**(3): p. 233-46.
